# Bivariate GWAS performed on rabbits divergently selected for intramuscular fat content reveals pleiotropic genomic regions and genes related to meat and carcass quality traits

**DOI:** 10.1186/s12711-025-00971-5

**Published:** 2025-07-11

**Authors:** Bolívar Samuel Sosa-Madrid, Agostina Zubiri-Gaitán, Noelia Ibañez-Escriche, Agustín Blasco, Pilar Hernández

**Affiliations:** Institute for Animal Science and Technology, Universitat Politècnica de València, 46022 Valencia, Spain

## Abstract

**Background:**

Meat quality plays an important economic role in the meat industry and livestock breeding programmes. Intramuscular fat content (IMF) is one of the main meat quality parameters and its genetic improvement has led breeders to investigate its genomic architecture and correlation with other relevant traits. Genetic markers associated with causal variants for these traits can be identified by bivariate analyses. In this study, we used two rabbit lines divergently selected for IMF to perform bivariate GWAS with the aim of detecting pleiotropic genomic regions between IMF and several weight, fat, and meat quality traits. Additionally, whole-genome sequencing data from these lines were used to identify potential causal variants associated with the genetic markers.

**Results:**

The main pleiotropic region was found on Oryctolagus cuniculus chromosome (OCC) 1 between 35.4 Mb and 38.2 Mb, explaining up to 2.66% of the IMF genetic variance and being associated with all traits analysed, except muscle lightness. In this region, the potentially causal variants found pointed to *PLIN2, SH3GL2, CNTLN*, and *BNC2* as the main candidate genes affecting the different weight, fat depots and meat quality traits. Other relevant pleiotropic regions found were those on OCC3 (148.94–150.89 Mb) and on OCC7 (27.07–28.44 Mb). The first was associated with all fat depot traits and explained the highest percentage of genetic variance, up to 10.90% for scapular fat. Several allelic variants were found in this region, all located in the novel gene *ENSOCUG00000000157* (orthologous to *ST3GAL1* in other species), involved in lipid metabolism, suggesting it as the main candidate affecting fat deposition. The region on OCC7 was associated with most meat quality traits and explained 8.48% of the genetic variance for pH. No allele variants were found to segregate differently between the lines in this region; however, it remains a promising region for future functional studies.

**Conclusions:**

Our results showed that bivariate models assuming pleiotropic effects are valuable tools to identify genomic regions simultaneously associated with IMF and several weight, fat and meat quality traits. Overall, our results provided relevant insights into the correlations and relationships between traits at the genomic level, together with potential functional mutations, which would be relevant for exploration in rabbit and other livestock breeding programmes.

**Supplementary Information:**

The online version contains supplementary material available at 10.1186/s12711-025-00971-5.

## Background

Meat quality has an increasingly important economic role in the meat industry and livestock breeding programmes [1], in which the ultimate goal is to meet consumers’ demands [2, 3]. It is a complex trait determined by multiple characteristics, including nutritional properties, organoleptic characteristics, and technological factors. The intramuscular fat content (IMF) is considered as one of the main meat quality parameters, since it affects several of the aforementioned characteristics, mainly the sensory properties such as flavour, juiciness and tenderness [3, 4]. Even though the relationship between IMF and these sensory traits varies between studies, higher IMF content is generally associated with greater tenderness and juiciness, and better flavour [5, 6]. However, increasing the IMF content can also affect the nutritional quality of meat, which is relevant for consumers’ acceptance, and it is also related to carcass quality traits, especially because of its positive correlation with carcass fat content [1, 2] which could lead to a deterioration of carcass quality.

The importance of IMF as a promising selection criterion has led to the development of several genetic experiments for example in beef cattle [7, 8], pigs [9, 10], chicken [11, 12], sheep [13], and rabbits [14, 15]. In fact, a successful divergent selection experiment for IMF was carried out in rabbits [14, 15], and genomic analyses have identified genomic regions and candidate genes associated with IMF [16, 17] and fatty acid composition [16]. Other traits were also measured in this selection experiment, such as carcass weight, fat depots, pH and colour parameter traits; and given their correlated responses and their moderate-to-high genetic correlations with IMF [14, 15], the selection for IMF could also affect causal variants and genes related to those traits. When a causal variant affects two or more traits in the same individual or species, this variant has a pleiotropic effect [18]. In breeding programmes, pleiotropic effects contribute to genetic covariances, together with linkage disequilibrium. Therefore, the discovery of genomic regions associated with favourable and unfavourable effects is important for understanding the genomic architecture of economically important traits, such as meat and carcass quality traits. Moreover, genetic markers linked to causal variants associated with IMF and other quality traits can be identified by bivariate analyses, which are assumed to have a greater power of detection [19, 20].

The aim of this study was to perform bivariate GWAS analyses to identify pleiotropic genomic regions associated with IMF and several carcass and meat quality traits. For this purpose, data from the two rabbit lines divergently selected for IMF were used and the results were compared with those obtained with univariate GWAS. Further enrichment, whole-genome sequencing analyses and visualization of genes on the associated genomic regions were also performed to integrate the results.

## Methods

### Animals and phenotypes

Samples from two rabbit lines divergently selected for intramuscular fat content (IMF) in the Longissimus thoracis et Lumborum (LTL) muscle were used to perform this study. This divergent selection experiment was carried out at the Universitat Politècnica de València and led to the creation of two lines: one with high-IMF content (H) and one with low-IMF content (L). Each line comprised 8–10 sires and 40–60 dams per generation; they were contemporarily reared under the same environmental and management conditions and were fed the same diet. The selection criterion was the IMF content measured at 9 weeks of age in two full sibs (one male and one female) of the first parity of each doe. Further details about the selection procedure can be found in Zomeño et al. and Martínez-Álvaro et al. [14, 15].

A total of 480 rabbits, 240 from the H line and 240 from the L line, from the 9th generation of selection were used in this study. The animals were slaughtered at 9 weeks of age using exsanguination after electrical stunning. A total of ten carcass traits (including weights, fat depots and colour), and five meat quality traits (including IMF, pH and colour) were recorded. The body weight (BW) of the rabbits was recorded before slaughter. After slaughter, the carcasses were chilled for 24 h at 4 ºC, and the chilled carcass weight (CCW) and liver weight (LW) were then recorded. Additionally, the reference carcass weight (RCW) was recorded as the weight of the carcass without the head, liver, lungs, thymus, oesophagus, heart, and kidneys [21]. The scapular (SF) and perirenal fat (PF) were excised and weighted, and the dissectible fat percentage (DF) was calculated as the sum of SF and PF, relative to the RCW. Finally, the carcass colour parameters lightness (CL*), redness (Ca*) and yellowness (Cb*) were measured on the surface of the fourth lumbar vertebra using a CR-400 Chroma Meter (Konica Minolta, Inc.).

To measure the meat quality traits, the LTL muscle was first dissected. The muscle pH was measured at the level of the fifth lumbar vertebra using a Crison pH-meter Basic + 20 (Crison Instruments, Barcelona, Spain), and the colour parameters lightness (LL*), redness (La*), and yellowness (Lb*) were measured at the seventh lumbar vertebra transversal section using the same colorimeter mentioned above. Finally, the muscle was minced and freeze dried, and the IMF content was quantified using near-infrared spectroscopy (NIRS; FOSS NIRSystems Inc., Hilleroed, Denmark), applying the equations developed by Zomeño et al. [22], and expressed as g IMF/100 g of muscle. Twenty percent of the samples were chemically analysed to test NIRS results.

Heritabilities, genetic correlations with IMF, and (co)variance components were estimated by fitting the bivariate animal model described by Martínez-Álvaro et al. [15] and using the TM software [23]. The dataset used comprised 2244 phenotypic records and 3676 animals in the pedigree from all generations.

### Genotypes and quality control

The DNA was extracted from samples of the obliquus abdominis muscle using standard procedures, and the genotyping was performed using the 200 K Affymetrix Axiom OrcunSNP array (ThermoFisher Scientific). A quality control analysis was performed by Axiom Analysis Suite v. 4.0.3 and only the SNPs with a call rate ≥ 0.95, a minor allele frequency (MAF) ≥ 0.05, and a known autosomal chromosome position according to OryCun2.0 assembly, were used in the association analyses. Moreover, animals with more than 3% of missing genotypes or that failed the Mendelian inheritance test were excluded; in the latter case, only the offspring failing the test was removed, while the parent was retained. The remaining missing genotypes were imputed using the software FImpute [24], inferring the haplotype using the complete genealogical information of the lines and also the genomic information of the parents (the 8th generation) obtained using the same aforementioned array. Finally, 477 animals (240 from the H and 237 from the L line) and 89,968 SNPs were retained for the association analyses. The vast majority of SNPs were removed because they were fixed in our populations, likely reflecting the fact that the SNP chip was not developed using rabbits from our specific lines, resulting in limited polymorphism at many loci.

### Statistical analyses

#### GWAS

Both univariate and bivariate GWAS were performed using Bayesian multiple-marker regression (BMMR) under the BayesB model. The bivariate GWAS included one carcass or meat quality trait as the first trait, and IMF as the second one. The model was defined as follows:

1 $$\left[\begin{array}{c}{\mathbf{y}}_{\mathbf 1}\\ {\mathbf{y}}_{\mathbf 2}\end{array}\right]=\left[\begin{array}{cc}{\mathbf{X}}_{\mathbf 1}& \mathbf{0}\\ \mathbf{0}& {\mathbf{X}}_{\mathbf 2}\end{array}\right]\left[\begin{array}{c}{\mathbf{b}}_{\mathbf 1}\\ {\mathbf{b}}_{\mathbf 2}\end{array}\right]+\left[\begin{array}{cc}{\mathbf{W}}_{1}& \mathbf{0}\\ \mathbf{0}& {\mathbf{W}}_{2}\end{array}\right]\left[\begin{array}{c}{\mathbf{c}}_{\mathbf 1}\\ {\mathbf{c}}_{\mathbf 2}\end{array}\right]+\left[\begin{array}{cc}{\mathbf{Z}}_{\mathbf 1}& \mathbf{0}\\ \mathbf{0}& {\mathbf{Z}}_{2}\end{array}\right]\left[\begin{array}{c}{\mathbf{u}}_{\mathbf 1}\\ {\mathbf{u}}_{\mathbf 2}\end{array}\right]+\sum_{{\varvec{j}}= \mathbf{1}}^{{\varvec{k}}}{\mathbf{z}}_{\mathbf{j}\mathbf{i}}{{\mathbf{D}}_{\mathbf{j}} \alpha }_{\text{j}} +\left[\begin{array}{c}{\mathbf{e}}_{\mathbf 1}\\ {\mathbf{e}}_{\mathbf 2}\end{array}\right],$$

where the subscript 1 refers to the analysed carcass or meat quality trait and the subscript 2 refers to the IMF trait; ${\mathbf{y}}_{\mathbf 1}$ and ${\mathbf{y}}_{\mathbf 2}$ are the phenotype vectors; ${\mathbf{b}}_{\mathbf 1}$ and ${\mathbf{b}}_{\mathbf 2}$ are the vectors of fixed effects, which included month (five levels), sex (two levels), and parity order (two levels); and ${\mathbf{c}}_{\mathbf 1}$ and ${\mathbf{c}}_{\mathbf 2}$ are the common litter random effects. Bearing in mind the missing heritability, a pedigree-based random polygenic effect represented by ${\mathbf{u}}_{\mathbf 1}$ and ${\mathbf{u}}_{\mathbf 2}$ was included in the model; ${\mathbf{X}}_{\mathbf 1}$, ${\mathbf{X}}_{\mathbf 2}$, ${\mathbf{W}}_{\mathbf 1}$, ${\mathbf{W}}_{\mathbf 2}$, ${\mathbf{Z}}_{\mathbf 1}$ and ${\mathbf{Z}}_{\mathbf 2}$ are the incidence matrices relating the corresponding effects to the phenotypes. $k$ is the total number of SNPs after quality control; ${\mathbf{z}}_{\mathbf{j}\mathbf{i}}$ is the vector containing the genotypic covariate for each SNP or locus $j$ (0, 1 or 2 reference alleles) for individual $i$; ${\alpha }_{j}$ is the random substitution effect for SNP $j$; ${\mathbf{D}}_{\mathbf{j}}$ is a diagonal matrix with elements diag (${\mathbf{D}}_{\mathbf{j}}$) = (${\delta }_{j1}$, ${\delta }_{j2}$), where ${\delta }_{j}$ is an indicator variable indicating if the marker effect of locus $j$ for each trait is zero or not. Further details regarding ${\delta }_{j}$ are explained below. Finally, ${\mathbf{e}}_{\mathbf 1}$ and ${\mathbf{e}}_{\mathbf 2}$ are the residual effects. The effects were assumed to be uncorrelated between each other.

The model was assumed to be conditionally distributed as a multivariate normal. Ordering the residuals by individual, the common litter random effects were distributed as $\mathbf{c} \sim {\rm N}(0,{\mathbf{I}}_{\mathbf{c}}\otimes \mathbf{C})$, and the polygenic random effects as $\mathbf{u} \sim {\rm N}(\mathbf{0} ,\mathbf{A}\otimes \mathbf{U})$; in which $\mathbf{C}$ and $\mathbf{U}$ are 2 × 2 (co)variances matrices between the two traits for the common litter ($\mathbf{C}$) and the polygenic ($\mathbf{U}$) effects; $\mathbf{A}$ is the full relationship matrix and ${\mathbf{I}}_{\mathbf{c}}$ is the identity matrix of the same order as the number of levels of common litter effects. Finally, ordering by individual, the residuals were distributed as $\mathbf{e} \sim N(\mathbf{0}, {\mathbf{I}}_{\mathbf{i}}\otimes \mathbf{R})$, where ${\mathbf{I}}_{\mathbf{i}}$ is an identity matrix with the same order as the number of individuals and $\mathbf{R}$ is the residual (co)variance matrix structured as follows:

2 $$\mathbf{R}=\boldsymbol{ }\left[\begin{array}{cc}{ \sigma }_{e1}^{2}& { \sigma }_{e\text{2,1}}\\ { \sigma }_{e\text{1,2}}& { \sigma }_{e2}^{2}\end{array}\right].$$

All (co)variance matrices were a priori assumed to follow an inverse Wishart distribution as described in Cheng et al. [20].

The BayesB model assumes that, at any given MCMC iteration, the majority of SNPs have zero effect on the phenotype (i.e., ${\delta }_{j}$ = 0, with a probability of π) and are therefore not included in the model [25]. In a bivariate GWAS, each SNP can affect any combination of traits. More specifically, there are four possible combinations, SNP $j$: (a) has zero effect on both traits (${\delta }_{j1}$=0, ${\delta }_{j2}$=0); (b) has an effect only on the first trait (${\delta }_{j1}$=1, ${\delta }_{j2}$=0); (c) has an effect only on the second trait (${\delta }_{j1}$=0, ${\delta }_{j2}$=1); or (d) has an effect on both traits (${\delta }_{j1}$=1, ${\delta }_{j2}$=1). After some exploratory analysis, performed following the methodology described in previous studies [16, 26], the joint probability (a) of SNPs with zero effect on both traits was set to 0.9982, which is the product of π1 and π2. The joint probability of the remaining combinations (b), (c) and (d) was proportionally distributed, each with a probability of 0.0006 (i.e., the remaining probability of 0.0018 divided by 3). The same model was used for the univariate GWAS of each carcass and meat quality trait, in which the π was set to 0.9988, as the one defined in the previous GWAS for IMF performed using these rabbit lines [16]. Finally, the effects of the SNPs that are not zero were assumed to be multinormally distributed as ${\rm N}(\mathbf{0}, {\mathbf{I}}_{\mathbf{j}}\otimes {\varvec{G}})$, where ${\mathbf{I}}_{\mathbf{j}}$ is an identity matrix with the same order as the number of SNPs and $\mathbf{G}$ is the (co)variance matrix of the random substitution effect structured as:

3 $$\mathbf{G}=\boldsymbol{ }\left[\begin{array}{cc}{ \sigma }_{\alpha j1}^{2}& { \sigma }_{\alpha j\text{2,1}}\\ { \sigma }_{\alpha j\text{1,2}}& { \sigma }_{\alpha j2}^{2}\end{array}\right],$$

which a priori assumed to follow an inverse Wishart distribution as described in Cheng et al. [20]. All effects were assumed to be independent from each other.

The marginal posterior distributions of the model parameters were estimated by Gibbs sampling, based on MCMC, consisting of 470,000 iterations, with a burn-in of 70,000. Only one sample every 40 iterations was saved to avoid the high correlation between consecutive samples. The prior (co)variance components were those previously estimated using the data from all generations, as explained above. Both univariate and bivariate GWAS were performed with the JWAS software [20]. The percentage of genetic variance was calculated by 1-Mb sliding genomic windows, allocated to the 21 autosomes, as this approach simplifies the tuning of the associated genomic regions. Genomic windows were considered to be associated with the analysed trait when they explained at least 0.75% of the genetic variance [19]. This threshold represents 14 times the expected genetic variance under an infinitesimal model (89,176 sliding genomic windows). The associated overlapping genomic windows were then grouped in only one genomic region to tune the position of the region in the rabbit genome. This sliding genomic window approach allows to better capture genomic regions associated with a trait than when using single marker regression analysis with Bayes factors [27]. As the selection increased the LD block between the IMF causal variants and the surrounding SNPs, the identification of associated genomic regions may gradually decrease, because the estimation of a causal variant effect would be shared among all surrounding SNPs that unlikely exceed a Bayes factor threshold. The posterior probability of association (PPA) for each SNP was then computed as the proportion of iterations in which the SNP was included in the model with a nonzero effect, relative to the total number of iterations. However, this parameter was not used as a threshold criterion given its dependence on π and the marker numbers in BayesB models, both at the chromosome- and genome-wide levels [26].

As the selection criterion was IMF, which is correlated with other meat and carcass quality traits, we hypothesised that a bivariate GWAS analysis [19, 28] would detect the regions affected by IMF selection as well as those moderately correlated with carcass and meat quality traits in rabbits. Thus, the genetic correlation between the two analysed traits for each sliding genomic window was estimated as the mean of the marginal posterior distribution of the correlation between the sampled breeding values for the two traits in the genomic region [19].

#### Identification of candidate genes and functional mutations

Candidate genes allocated in the associated genomic windows were obtained from the Ensembl Genes 111 database, using the Oryctolagus cuniculus as the reference genome (OryCun 2.0.) [29]. The biological functions and pathways of the genes were retrieved from the Database for Annotation, Visualization and Integrated Discovery (DAVID) v.6.8 [30]. The genes related to retrieved pathways were also investigated using the gene ontology (GO) [31].

Whole-genome sequencing (WGS) from the 20 breeding males of the 8th generation, corresponding to the sires of the animals used in the GWAS, was performed to identify the genomic variants present in the associated genomic regions that segregated differently between the lines, indicating possible functional mutations. The sequencing, pre-processing, and variant calling were performed at the Centro Nacional de Análisis Genómico (CNAG-CRG Barcelona, Spain). Briefly, sequencing was performed using Illumina Technology; and the WGS data was pre-processed following the methodology described by Wright, et al. [32]. Illumina adapters and low-quality ends were removed using Trimmomatic v0.39 [33] and the BWA-MEM algorithm was then applied to align the reads to the OryCun v2.0 reference genome [34]. Variant calling was performed applying the GATK Best Practices pipeline [35], using HaplotypeCaller and GenotypeGVCF, to finally obtain the VCF files. Variants were annotated using the snpEff software [36] and only those SNVs and INDELs with moderate to high impact were considered, which included: missense and frameshift variants, start lost, stop gained, conservative in frame deletions and insertions [37]. Finally, we identified as relevant (1) the allele variants that were fixed in one line and segregating at an allele frequency lower than 0.5 in the other, as these suggest directional selection or lineage-specific fixation, and (2) the variants whose alleles were segregating at opposite frequencies between lines (greater than 0.65), indicating substantial differentiation. These thresholds were chosen to balance stringency with sensitivity, allowing us to capture biologically meaningful divergence while minimizing noise from minor frequency shifts.

## Results

Descriptive statistics of the studied traits before the correction for systematic effects are shown in Table 1, while the differences between lines can be found in previous studies [15, 38]. Heritabilities, genetic correlations with IMF, and the common litter random effects are shown in Table 2.

**Table 1:** Descriptive statistics of carcass and meat quality traits in the 9th generation of divergent selection experiment

Trait	High-IMF line					Low-IMF line				
	NumA^a^	Mean	SD^b^	Range^c^	CV%	NumA^a^	Mean	SD^b^	Range^c^	CV%
IMF (g/100 g)	240	1.32	0.21	[0.87, 1.94]	15.85	237	0.80	0.06	[0.63, 1.08]	8.01
BW (g)	240	1828	140	[1502, 2096]	7.66	237	1777	144	[1520, 2168]	8.13
CCW (g)	240	975	104	[703, 1219]	10.68	237	933	116	[709, 1312]	12.46
RCW (g)	240	848	79	[636, 1004]	9.28	235	808	89	[639, 1088]	10.96
LW (g)	237	57.81	8.97	[39.85, 81.07]	15.52	235	51.24	7.53	[37.09, 75.83]	14.69
SF (g)	234	4.60	1.24	[1.5, 8.52]	26.95	236	3.60	1.48	[0.96, 10.54]	41.01
PF (g)	239	11.22	3.53	[1.75, 21.58]	31.49	237	5.99	2.89	[1.18, 16.19]	48.28
DF (%)	234	1.85	0.44	[0.48, 3.04]	23.93	234	1.16	0.42	[0.36, 2.58]	36.22
CL*	239	51.65	1.89	[46.08, 56.72]	3.65	236	51.68	1.92	[46.79, 58.77]	3.72
Ca*	239	1.98	0.68	[0.65, 4.61]	34.33	236	2.04	0.79	[0.69, 6.09]	38.79
Cb*	239	− 1.99	1.41	[− 5.74, 1.94]	NA	236	− 2.18	1.69	[− 7.46, 2.28]	NA
pH	239	5.72	0.10	[5.38, 5.97]	1.73	237	5.73	0.10	[5.37, 6.05]	1.78
LL*	240	50.63	1.82	[45.55, 57.08]	3.60	237	50.72	2.19	[44.87, 58.34]	4.33
La*	240	2.24	0.92	[− 0.14, 5.11]	NA	237	2.28	1.06	[0.23, 7.53]	46.60
Lb*	240	1.69	0.66	[− 0.83, 3.72]	NA	237	1.62	0.72	[− 1.95, 3.91]	NA

NumA^a^: number of animals after quality control; SD^b^: standard deviation; Range^c^: includes the minimum (left) and the maximum values

*BW:* body weight, *CCW*: commercial carcass weight, *RCW:* reference carcass weight, *LW*: liver weight, *SF*: scapular fat, *PF*: perirenal fat, *DF*: dissectible fat percentage,  CL*: carcass lightness, Ca*: carcass redness ,  Cb*: carcass yellowness, LL*: muscle lightness, La*: muscle redness, Lb*: muscle yellowness

**Table 2:** Heritabilities of carcass and meat quality traits and their genetic correlations with intramuscular fat content

Trait	Heritability		Genetic correlation			C^2^ of common litter effect^c^	
	Mean	HPD_95%_^a^	Mean	HPD_95%_^a^	P_0_^b^	Mean	HPD_95%_^a^
Carcass-weight traits							
BW	0.28	[0.13, 0.43]	0.06	[− 0.24, 0.34]	0.67	0.28	[0.22, 0.35]
CCW	0.26	[0.13, 0.41]	0.09	[− 0.20, 0.39]	0.72	0.29	[0.22, 0.36]
RCW	0.30	[0.15, 0.46]	0.06	[− 0.23, 0.35]	0.65	0.27	[0.21, 0.34]
LW	0.38	[0.15, 0.64]	0.18	[− 0.16, 0.52]	0.84	0.24	[0.14, 0.33]
Fat depot traits							
SF	0.43	[0.29, 0.57]	0.28	[0.04, 0.51]	0.98	0.14	[0.08, 0.20]
PF	0.58	[0.43, 0.75]	0.31	[0.09, 0.53]	0.99	0.15	[0.09, 0.21]
DF	0.66	[0.51, 0.80]	0.34	[0.12, 0.55]	1.00	0.11	[0.06, 0.17]
Meat quality traits							
IMF	0.49	[0.35, 0.63]	–	–	–	0.17	[0.11, 0.23]
CL*	0.18	[0.08, 0.31]	− 0.11	[− 0.41, 0.21]	0.75	0.31	[0.24, 0.37]
Ca*	0.36	[0.20, 0.51]	0.00	[− 0.27, 0.28]	0.50	0.19	[0.13, 0.26]
Cb*	0.11	[0.01, 0.22]	0.11	[− 0.27, 0.47]	0.73	0.28	[0.21, 0.36]
pH	0.07	[0.00, 0.14]	0.10	[− 0.25, 0.46]	0.71	0.32	[0.26, 0.37]
LL*	0.23	[0.09, 0.37]	0.00	[− 0.29, 0.32]	0.51	0.33	[0.26, 0.40]
La*	0.32	[0.19, 0.47]	0.09	[− 0.19, 0.35]	0.73	0.13	[0.08, 0.19]
Lb*	0.20	[0.09, 0.32]	0.27	[− 0.01, 0.53]	0.96	0.21	[0.15, 0.27]

*IMF:* intramuscular fat content, *BW:* body weight, *CCW:* commercial carcass weight, *RCW:* reference carcass weight, *LW:* liver weight, *SF:* scapular fat, *PF:* perirenal fat, *DF:* dissectible carcass fat percentage, CL*: carcass lightness, Ca*: carcass redness, Cb*: carcass yellowness, LL*: muscle lightness, La*: muscle redness, Lb*: muscle yellowness

^a^HPD95%: the highest posterior density interval at 95%

^b^P0: probability of the correlation being greater than zero when positive or lower than zero when negative

^c^C^2^ of common litter effect: proportion of phenotypic variance explained by the common litter effect

Regarding the GWAS results, there was a general increase in the number of associated SNPs detected when performing the bivariate GWAS than when performing the univariate one for each trait considered (Tables 3, 4, and 5). As mentioned above, in this study we focussed on the genomic regions found to be associated by genomic window analysis, as only one or few SNPs exceeding a SNP threshold in the single marker regression method are likely to be false positives, even more so if the SNP was not found to be associated by genomic window analysis. The associated overlapping genomic windows have been grouped in only one genomic region to tune the position of the region in the rabbit genome. Therein, this tuned region is defined as associated genomic region. For simplicity and better visualization, the results are presented graphically or in tables based on overlapping sliding windows across GWAS analyses, i.e., the associated genomic regions across all traits have been clustered into: (a) weight traits: BW, CCW, RCW and LW; (b) fat depot traits: PF, SF and DF; and (c) meat quality traits: all colour parameter traits and pH. The bivariate GWAS allowed to detect genomic regions associated with both traits included in the analysis (i.e., regions with pleiotropic effects; Table 6), but also genomic regions associated with only one of the traits (Tables 3, 4, and 5).

**Table 3:** Genomic regions associated with weight traits in rabbits

OCC	Position in genome		Trait	P_Gen_Var UNIVAR	(PPA) UNIVAR	P_Gen_Var BIVAR	(PPA) BIVAR
	Start	End					
1	28901821	35233047	CCW	2.79	(0.3899)	2.40	(0.5195)
			RCW	1.98	(0.3574)	1.93	(0.4582)
			BW	2.40	(0.4551)	2.13	(0.3860)
			LW	2.58	(0.4140)	1.56	(0.3739)
	35421203	38061945	BW	–	–	2.56	(0.4585)
			CCW	–	–	2.50	(0.6078)
			LW	–	–	3.36	(0.4716)
			RCW	–	–	2.29	(0.5413)
	42411932	44790700	RCW	3.42	(0.4189)	2.18	(0.3882)
			CCW	2.28	(0.3165)	1.50	(0.3334)
			BW	0.83	(0.2198)	–	–
2	139299216	140642584	LW	0.85	(0.3537)	–	–
5	9779841	11429918	LW	1.05	(0.3703)	0.83	(0.3405)
6	2242936	3567876	LW	0.88	(0.3266)	–	–
7	110543262	112253533	CCW	0.96	(0.2938)	0.96	(0.3742)
			RCW	1.22	(0.3369)	1.15	(0.3667)
	156958947	158112218	BW	–	–	1.02	(0.2871)
			CCW	–	–	0.75	(0.2780)
	158765428	160790627	BW	0.93	(0.2659)	1.53	(0.3596)
			CCW	0.88	(0.26830	1.48	(0.4290)
			RCW	0.81	(0.2694)	1.15	(0.3726)
9	74758537	77293167	LW	1.27	(0.3906)	0.96	(0.3367)
10	16545201	17708416	BW	–	–	0.83	(0.2814)
12	30386038	33742686	LW	3.69	(0.4474)	2.62	(0.4278)
13	29328268	30685073	BW	0.96	(0.2676)	–	–
			RCW	0.96	(0.2766)	–	–
14	70440101	72889757	BW	0.98	(0.2296)	2.29	(0.3851)
	134303242	136330626	CCW	1.50	(0.3432)	1.91	(0.5745)
			RCW	1.36	(0.3808)	1.83	(0.5166)
			BW	0.80	(0.2834)	1.04	(0.3514)
15	10831534	12836884	LW	1.71	(0.4325)	1.29	(0.3986)
			BW	1.12	(0.3081)	–	–
17	9861967	12054383	CCW	0.76	(0.2815)	3.42	(0.6866)
			RCW	–	–	2.37	(0.5925)
			BW	–	–	1.29	(0.4535)
19	28333037	30004320	BW	–	–	1.04	(0.3294)
	30099870	32818846	LW	1.26	(0.3997)	1.51	(0.4418)
			BW	1.52	(0.3747)	2.06	(0.4210)
			CCW	1.12	(0.3134)	1.89	(0.5585)
			RCW	0.93	(0.3010)	1.44	(0.4459)
20	19038679	20588204	BW	3.21	(0.4975)	2.55	(0.4620)
			CCW	2.20	(0.4175)	2.27	(0.6231)
			RCW	2.27	(0.4465)	2.08	(0.5655)

OCC: Oryctolagus cuniculus chromosome. P_Gen_Var: percentage of genetic variance accounted for the associated genomic region; (PPA) = posterior probability of association or inclusion in the model; UNIVAR: univariate model analysis; BIVAR: univariate model analysis between this trait and intramuscular fat content. “–“ = non associated genomic region for this model as the genomic region does not exceed the genomic window threshold (0.75). *BW*: body weight; *CCW*: commercial carcass weight; *RCW*: reference carcass weight; *LW:* liver weight

**Table 4:** Genomic regions associated with fat depot traits in rabbits

OCC	Position in genome		Trait	P_Gen_Var UNIVAR	(PPA) UNIVAR	P_Gen_Var BIVAR	(PPA) BIVAR
	Start	End					
1	27570818	28755852	DF	0.83	(0.44)	–	–
	30428887	32483725	PF	1.65	(0.6388)	–	–
	33710991	38203204	PF	1.24	(0.4977)	4.35	(0.7668)
			DF	0.80	(0.4684)	3.14	(0.7470)
			SF	–	–	2.31	(0.8742)
	174113797	175711375	DF	–	–	0.85	(0.7048)
			PF	–	–	0.78	(0.6847)
3	138874133	140152676	PF	0.77	(0.3838)	1.00	(0.4973)
	148788842	151571092	SF	10.32	(0.9347)	10.90	(0.9687)
			DF	4.54	(0.9361)	5.13	(0.9589)
			PF	3.15	(0.8676)	3.50	(0.9247)
4	71082447	72639613	SF	–	–	0.77	(0.5960)
5	10154337	11665709	DF	0.91	(0.7625)	–	–
	29840263	31819891	PF	–	–	1.41	(0.8708)
			DF	–	–	1.06	(0.8357)
6	3936191	6108224	DF	3.84	(0.9663)	3.14	(0.9573)
			PF	1.79	(0.7989)	1.15	(0.6995)
			SF	1.17	(0.6058)	–	–
9	15473709	17491458	SF	1.24	(0.7700)	1.63	(0.7409)
			DF	1.13	(0.7814)	0.89	(0.6678)
12	42683826	44688701	PF	1.74	(0.6513)	–	–
13	100353890	101950026	DF	–	–	0.86	(0.6097)
	139448688	141261644	DF	1.46	(0.7405)	–	–
			SF	1.70	(0.7965)	–	–
14	42451260	43654926	SF	–	–	0.80	(0.4723)
	134489752	135928030	SF	–	–	0.86	(0.4756)
17	10061927	11798813	PF	–	–	0.87	(0.7564)
19	30530951	33065887	PF	1.41	(0.7623)	2.32	(0.8118)
			DF	0.85	(0.6803)	1.40	(0.7668)
	54592191	56054881	SF	–	–	0.86	(0.4305)

OCC: Oryctolagus cuniculus chromosome. P_Gen_Var: percentage of genetic variance accounted for the associated genomic region; (PPA) = posterior probability of association or inclusion in the model; UNIVAR: univariate model analysis; BIVAR: bivariate model analysis between this trait and intramuscular fat content. “–“ = non associated genomic region for this model as the genomic region does not exceed the genomic window threshold (0.75). *SF*: scapular fat; *PF*: perirenal fat; *DF*: dissectible carcass fat percentage

**Table 5:** Genomic regions associated with meat quality traits in rabbits

OCC	Position in genome		Trait	P_Gen_Var UNIVAR	(PPA) UNIVAR	P_Gen_Var BIVAR	(PPA) BIVAR
	Start	End					
1	35421203	37880078	La*	–	–	1.57	(0.3732)
			pH	–	–	1.65	(0.5932)
			Ca*	–	–	1.46	(0.4990)
			Cb*	–	–	1.17	(0.3757)
			CL*	–	–	1.04	(0.3486)
			Lb*	–	–	0.76	(0.3041)
	38786430	40524065	La*	0.84	(0.2501)	0.78	(0.2734)
	85332793	86332793	La*	0.75	(0.2193)	–	–
	120340193	122103410	pH	–	–	0.95	(0.5754)
			Cb*	–	–	0.90	(0.3920)
2	19814073	20883718	LL*	0.90	(0.2862)	–	–
	152094935	153292895	Ca*	0.84	(0.2522)	0.76	(0.2584)
3	34588926	36743147	Lb*	1.97	(0.2419)	1.67	(0.2756)
	38625731	41531359	Lb*	1.57	(0.241)	1.27	(0.2699)
	123774002	125777015	Lb*	–	–	0.95	(0.2456)
	133620124	135530513	Lb*	–	–	0.99	(0.2108)
	138874133	140306258	CL*	–	–	1.46	(0.2849)
			Lb*	1.09	(0.2461)	–	–
4	62983442	64506834	LL*	0.87	(0.2621)	–	–
	66806370	68324187	Ca*	0.82	(0.2675)	–	–
	70693711	72818170	Ca*	–	–	2.98	(0.5346)
			pH	–	–	0.93	(0.4124)
			Lb*	–	–	0.83	(0.3446)
	74338850	75611556	LL*	0.89	(0.2443)	–	–
5	7298347	8860898	Lb*	–	–	0.80	(0.2807)
	15837999	19050703	Ca*	2.97	(0.3919)	2.24	(0.4636)
	20180124	22111503	CL*	1.33	(0.2612)	0.87	(0.233)
7	5452	1979376	Lb*	1.71	(0.2535)	1.87	(0.3275)
	2518414	4805150	Lb*	1.09	(0.2181)	1.18	(0.2521)
	8256520	9284111	Lb*	0.76	(0.2166)	–	–
	26743882	28900797	pH	–	–	8.48	(0.6734)
			Ca*	–	–	1.13	(0.482)
			Cb*	–	–	0.79	(0.3531)
			La*	–	–	0.80	(0.3402)
	45455815	47590522	La*	1.65	(0.3593)	1.23	(0.3227)
			Ca*	1.11	(0.354)	–	–
	69048438	70596609	CL*	0.84	(0.2273)	–	–
	72316729	73316729	Ca*	–	–	0.75	(0.2735)
	96996061	98552759	La*	0.86	(0.2602)	–	–
	167220424	168333021	Cb*	0.81	(0.1563)	–	–
8	1663933	3568211	Ca*	1.12	(0.3036)	0.97	(0.2968)
	68365260	70147889	La*	1.04	(0.2376)	1.15	(0.2807)
	99133029	100490608	LL*	0.85	(0.2433)	–	–
9	15140254	16993305	LL*	1.02	(0.2416)	–	–
	67289669	70258747	Lb*	–	–	2.57	(0.3983)
			Ca*	–	–	1.81	(0.3949)
			La*	–	–	1.84	(0.3640)
			pH	–	–	1.44	(0.3659)
			Cb*	–	–	1.02	(0.3564)
	70807676	71835923	Ca*	–	–	0.86	(0.2473)
	85041985	88396569	CL*	2.06	(0.2898)	1.45	(0.2803)
	90435608	91658263	CL*	0.93	(0.2435)	–	–
10	17680826	19827740	La*	2.12	(0.3259)	2.53	(0.3529)
11	83081198	85377090	LL*	2.75	(0.3939)	–	–
12	47446494	48928659	CL*	0.94	(0.2373)	–	–
	52181759	54177785	LL*	1.30	(0.3082)	–	–
	111962950	113947302	CL*	–	–	1.05	(0.2634)
13	66873358	68737843	LL*	1.43	(0.2941)	–	–
	72861641	75624854	LL*	2.92	(0.3614)	1.02	(0.547)
	93880669	95590867	Cb*	–	–	0.88	(0.3942)
	122014443	123999114	Ca*	1.44	(0.3146)	1.41	(0.3205)
	128853115	130283007	La*	0.79	(0.2233)	–	–
14	42146089	44087078	La*	1.05	(0.2714)	0.77	(0.2507)
15	4496821	5673569	LL*	0.77	(0.2669)	–	–
	6726456	9405305	Lb*	–	–	1.21	(0.3797)
	19416243	20467908	LL*	0.76	(0.2232)	–	–
	22789228	24896386	Ca*	1.94	(0.3850)	1.73	(0.4783)
16	60801609	62850595	La*	1.20	(0.2944)	1.53	(0.3182)
	82755837	84474303	Lb*	–	–	1.95	(0.3260)
			La*	–	–	1.02	(0.2994)
17	4535591	5969924	La*	–	–	0.99	(0.2948)
			Lb*	–	–	1.87	(0.3216)
	34753835	36798911	Cb*	1.41	(0.2244)	0.90	(0.2394)
	36844016	40530309	Cb*	1.53	(0.2412)	1.09	(0.2843)
	55656893	57571280	Ca*	0.93	(0.2956)	0.88	(0.3137)
18	34695954	36361523	Ca*	1.08	(0.3294)	0.80	(0.287)
	60905831	62116156	Cb*	0.76	(0.1676)	–	–
	62484401	63886847	Cb*	0.84	(0.1377)	–	–
19	33967423	35909499	Lb*	–	–	1.18	(0.2739)
	53978774	56003104	LL*	1.44	(0.2859)	–	–
			Lb*	0.83	(0.2019)	–	–
20	22699320	26339396	La*	0.93	(0.2283)	0.78	(0.1708)

OCC: Oryctolagus cuniculus chromosome. P_Gen_Var: percentage of genetic variance accounted for the associated genomic region; (PPA) = posterior probability of association or inclusion in the model; UNIVAR: univariate model analysis; BIVAR: bivariate model analysis between this trait and intramuscular fat content. “–“ = non associated genomic region for this model as the genomic region does not exceed the genomic window threshold (0.75). CL*: carcass lightness; Ca*: carcass redness; Cb*: carcass yellowness; LL*: muscle lightness; La*: muscle redness, Lb*: muscle yellowness

**Table 6:** Genomic correlations between regions associated with intramuscular fat (IMF) and carcass-weight, fat depot and meat quality traits in rabbits

OCC	Genomic regions associated only with IMF					
	IMF-assGR (Mb)	IMF-P_Gen_Var	IMF-(PPA)	Traits (bivariate analysis)	Highest GenCor	Correlation SD
1	120.28–122.19	1.16	(0.7555)	IMF & Ca*	0.01	0.5200
		1.20	(0.7717)	IMF & Lb*	0.10	0.4844
		1.05	(0.7256)	IMF & La*	0.13	0.5319
		0.92	(0.6839)	IMF & BW	0.15	0.5285
		0.90	(0.6691)	IMF & RCW	0.06	0.5062
		0.81	(0.637)	IMF & CCW	0.07	0.4783
		0.76	(0.7145)	IMF & DF	0.26	0.4627
		0.76	(0.808)	IMF& SF	0.29	0.4894
	142.26–143.41	0.79	(0.7803)	IMF & DF	− 0.003	0.3877
9	68.31–69.37	0.79	(0.5768)	IMF & CCW	0.10	0.4678
		0.77	(0.5622)	IMF & RCW	0.07	0.4569
13	93.68–95.61	1.08	(0.8501)	IMF & PF	0.08	0.4036
		0.80	(0.6536)	IMF & RCW	0.19	0.5206
		0.76	(0.6408)	IMF & CCW	0.18	0.5346
		0.78	(0.7200)	IMF & Lb*	0.10	0.4371
16	83.08–84.47	1.03	(0.5627)	IMF & LW	−0.04	0.4259
17	4.54–5.80	0.98	(0.5519)	IMF & LW	−0.04	0.4129

Genomic regions associated with both IMF and specific trait							
OCC	IMF- assGR (Mb)	IMF-P_Gen_Var	IMF-(PPA)	Trait	Position in the genome (Mb)	Highest GenCor	Correlation SD
1	35.42–37.70	2.00	(0.6844)	BW	35.42–38.06	0.29	0.5699
	35.42–37.63	1.78	(0.6484)	CCW		0.31	0.5561
	35.45–37.54	1.59	(0.6190)	LW		0.40	0.5359
	35.45–37.67	1.77	(0.6530)	RCW		0.29	0.5596
	35.48–37.71	1.82	(0.7911)	PF	33.71–38.20	0.60	0.5034
	35.54–37.90	1.65	(0.7675)	DF		0.58	0.5049
	35.42–37.62	2.00	(0.8923)	SF		0.40	0.5406
	35.42–37.80	2.38	(0.7309)	La*	35.42–37.88	− 0.17	0.5646
	35.42–37.78	2.23	(0.7389)	pH		0.10	0.5825
	35.42–37.80	2.44	(0.7500)	Ca*		− 0.17	0.5622
	35.42–37.80	2.19	(0.7241)	Cb*		0.02	0.4827
	35.54–37.71	1.75	(0.6837)	CL*		− 0.09	0.5508
	35.42–37.78	2.66	(0.7585)	Lb*		0.02	0.3730
	120.30–122.18	1.10	(0.7554)	pH	120.34–122.10	− 0.08	0.5727
	120.30–122.16	1.08	(0.7296)	Cb*		0.19	0.5805
3	148.94–150.89	1.21	(0.9291)	SF	148.79–151.57	0.92	0.2746
7	27.07–28.44	0.75	(0.7599)	pH	26.74- 28.90	0.63	0.5144
9	68.31–69.36	0.77	(0.6397)	Lb*	67.29–70.26	0.34	0.5044
	67.93–69.37	0.81	(0.6128)	pH		− 0.15	0.4935
	67.93–69.37	0.79	(0.5951)	Cb*		0.17	0.4952
13	93.88–95.57	0.84	(0.7000)	Cb*	93.88–95.59	0.21	0.5546

OCC: Oryctolagus cuniculus chromosome. P_Gen_Var: percentage of genetic variance accounted for the associated genomic region; (PPA) = posterior probability of association or inclusion in the model; UNIVAR: univariate model analysis; BIVAR: bivariate model analysis between this trait and intramuscular fat content; Highest GenCor: Highest genetic correlation estimate amongst all associated consecutive genomic windows that belong to the genomic region. Correlation SD: correlation standard deviation of the highest genetic correlation estimate *BW*: body weight; *CCW*: commercial carcass weight; *RCW*: reference carcass weight; *LW*: liver weight; *SF*: scapular fat; *PF*: perirenal fat; *DF*: dissectible carcass fat percentage; CL*: carcass lightness, Ca*: carcass redness, Cb*: carcass yellowness; LL*: muscle lightness, La*: muscle redness, Lb*: muscle yellowness

### Weight traits

The bivariate analyses allowed us to identify three genomic regions associated with all weight traits (BW, CCW, RCW and LW); two located on Oryctolagus cuniculus chromosome (OCC) 1 and one on OCC19 (Table 3). In addition, genomic regions found on OCC 7, 14, 17 and 20 were associated with BW, CCW, and RCW, as expected due to the high correlation between these traits. From the aforementioned regions, those located at 35.42–38.06 Mb on OCC1 and at 9.86–12.05 Mb on OCC17 were not detected in the corresponding univariate analyses, likely due to the low percentage of genetic variance that they explained. On the other hand, the associated genomic regions explaining the highest percentage of genetic variance matched between the univariate and bivariate model analyses. This was the case for the regions located at 28.90–35.23 Mb on OCC1 (from 1.56 to 2.79% of genetic variance explained considering both univariate and bivariate results), and 30.10–32.82 Mb on OCC19 (from 0.93 to 2.06%), associated with the four traits; the region located at 19.04–20.59 Mb on OCC20 (from 2.08 to 3.21%) associated with BW, CCW and RCW traits; and the region located at 30.38–33.74 Mb on OCC12 (2.62% in the univariate GWAS and 3.69% in the bivariate GWAS) associated with LW (Table 3).

### Fat depot traits

The genomic regions associated with fat depot traits presented the highest percentages of explained genetic variance of all traits analysed in the current study (Table 4). The regions located at 33.71–38.20 Mb on OCC1 and at 148.79–151.57 Mb on OCC3 were related to all fat depot traits (PF, SF and DF), and were also the regions explaining the highest percentage of genetic variance (from 0.80 to 4.35% for the region on OCC1 and from 3.15 to 10.90% for the region on OCC3, considering both univariate and bivariate results). The highest percentage of genetic variance explained on OCC3 corresponded to the SF (10.32% in the univariate GWAS and 10.90% in the bivariate GWAS), reaching up to 0.97 of PPA. Another relevant genomic region was that located at 3.94–6.10 Mb on OCC6, which accounted for up to 3.14% of genetic variance for DF.

The results of weight and fat depot traits showed two common regions associated with most traits: the one located at 33.71–38.20 Mb on OCC1 (0.80–4.35%) and at 30.53–33.06 Mb on OCC19 (0.85–2.32%), suggesting that these genomic regions have relevant effects for both sets of traits.

### Meat quality traits

The genomic regions associated with meat quality and carcass colour traits are shown in Table 5. These associated genomic regions were scattered across the whole rabbit genome, with the only exception of OCC21. Several genomic regions were clearly associated when the bivariate GWAS analysis was performed, most likely due to their relationship and association with IMF, whereas no associations were found in the univariate GWAS. Among these regions, those located at 35.42–37.88 Mb on OCC1, 26.74–28.90 Mb on OCC7 and 67.28–70.26 Mb on OCC9 stand out because they were associated with most of the studied traits. The region on OCC1 presented association with pH (1.65% of genetic variance explained), La* (1.57%), Lb* (0.76%), Ca* (1.46%), Cb* (1.17%), and CL* (1.04%), and it overlapped with the associated region found for weight and fat depot traits. On the other hand, the region on OCC7 was associated with pH (8.48%), Ca* (1.13%), Cb* (0.79%) and La* (0.80%), while that in OCC9 was associated with Lb* (2.57%), Ca* (1.81%), La* (1.84%), pH (1.44%) and Cb* (1.02%). The regions located at 120.34–122.10 Mb on OCC1 (0.90–0.95%), 70.69–72.82 Mb on OCC4 (0.83–2.98%), 82.76–84.47 Mb on OCC16 (1.02–1.95%), and 45.36–59.70 Mb on OCC17 (0.99–1.87%) were other associated genomic regions identified only with bivariate model analysis.

### Pleiotropic genomics regions associated with intramuscular fat

Table 6 shows the genomic regions found in all bivariate analyses that were associated only with IMF and those associated with both traits (i.e., pleiotropic regions).

Associated genomic regions with pleiotropic effects were found on OCC1, OCC3, OCC7, OCC9 and OCC13. The most important region was the one located at 35.42–37.90 Mb on OCC1, which explained up to 2.66% of the genetic variance of IMF and, as mentioned before, it was also associated with all analysed traits with the only exception of LL*. Other regions to highlight were those located at 148.94–150.89 Mb on OCC3 and 27.07–28.44 Mb on OCC7. The first one was the most relevant region for SF and explained 1.21% of the genetic variance of IMF, while the second one was remarkably important for pH and explained 0.75% of the genetic variance of IMF. These pleiotropic regions were supported by the high genomic correlations between the traits for each specific region (Table 6). However, it is worth mentioning that the magnitude of the genomic correlation of the associated regions depends on the trait analysed and is not necessarily related to the association. For example, in the bivariate analysis for the colour trait Cb*, the region 120–122 Mb on OCC1 presented association with both traits and had a genomic correlation of 0.19, whereas in the bivariate analysis for DF this same region had a higher genomic correlation (0.26) but was only associated with IMF and not with DF.

### Gene search and identification of functional mutations

The associated genomic regions found in all analyses comprised 1687 genes: 500 genes for weight traits, 385 genes for fat depot traits, and 1005 genes for meat quality traits (see Additional file 1: Table S1).

The WGS performed on the sires allowed to identify a large number of allele variants present in the associated genomic regions for each set of traits (see Additional file 2: Table S2). From those, we kept (1) the allele variants that were fixed in one line and segregating at an allele frequency lower than 0.5 in the other, and (2) the variants whose alleles were segregating at opposite frequencies (greater than 0.65) between lines. We finally detected 167 variants (located in 69 genes) in the genomic regions associated to weight traits (see Additional file 3: Table S3); 79 variants (located in 28 genes) in the genomic regions associated to fat traits (see Additional file 4: Table S4); and 134 variants (located in 46 genes) in the genomic regions associated to meat quality traits (see Additional file 5: Table S5).

When analysing the main pleiotropic regions highlighted in the previous section, there were 13 variants detected: 7 on the pleiotropic region of OCC1 (associated to almost all traits) and located in genes *SH3GL2*, *CNTLN*, and *BNC2*; and 6 on the pleiotropic region of OCC3 (associated to all fat traits and explaining a large percentage of genetic variance), all located in the *ENSOCUG00000000157* gene. Unfortunately, we did not detect any relevant variant on the pleiotropic region of OCC7 (explaining a large percentage of the genetic variance of pH) under the assumed parameters.

## Discussion

Understanding how selection for IMF affects the correlated traits is important for making accurate decisions in livestock breeding programmes. Causal variants can affect more than one trait, so the study of their pleiotropic contributions could be relevant in this sense. In fact, bivariate GWAS of two highly correlated traits can increase the power to detect associations on both studied traits [19, 20, 28]. The detection of variants and genomic regions associated with a particular trait also depends on the model and its prior parameters. For instance, novel genomic regions associated with IMF were discovered in these lines when a maternal genetic effect was used in the model [39]. On the other hand, the priors can influence the results of the various “Bayesian alphabet” GWAS methods, especially when the number of unknown parameters (p) exceeds the sample size (n), which is typical in genomic analyses [25, 26, 40]. However, with largely polygenic traits, the identification of genomic regions or variants using regression methods based on whole genome can be difficult even when n ≈ p. A GWAS for body weight at 35 days performed in broilers using an extremely large sample (137,000 genotyped individuals) could only identify 25 genomic regions that together explained ~ 30% of the genetic variance [41]. In practice, most common Bayesian methods for GWAS, such as B, C, C-π and R, are able to separate the “noise” from those genomic variants with relative relevance to a trait, thus improving the detection of variants and genomic regions associated [20]. Therefore, in this study we assumed that the genomic regions detected could be considered as associated but probably explain a slightly lower variance than those found. Furthermore, given that pleiotropy is being increasingly considered in genomic evaluations, bivariate GWAS would be a relevant genetic analysis to explore in livestock breeding programmes.

**Fig. 1:**
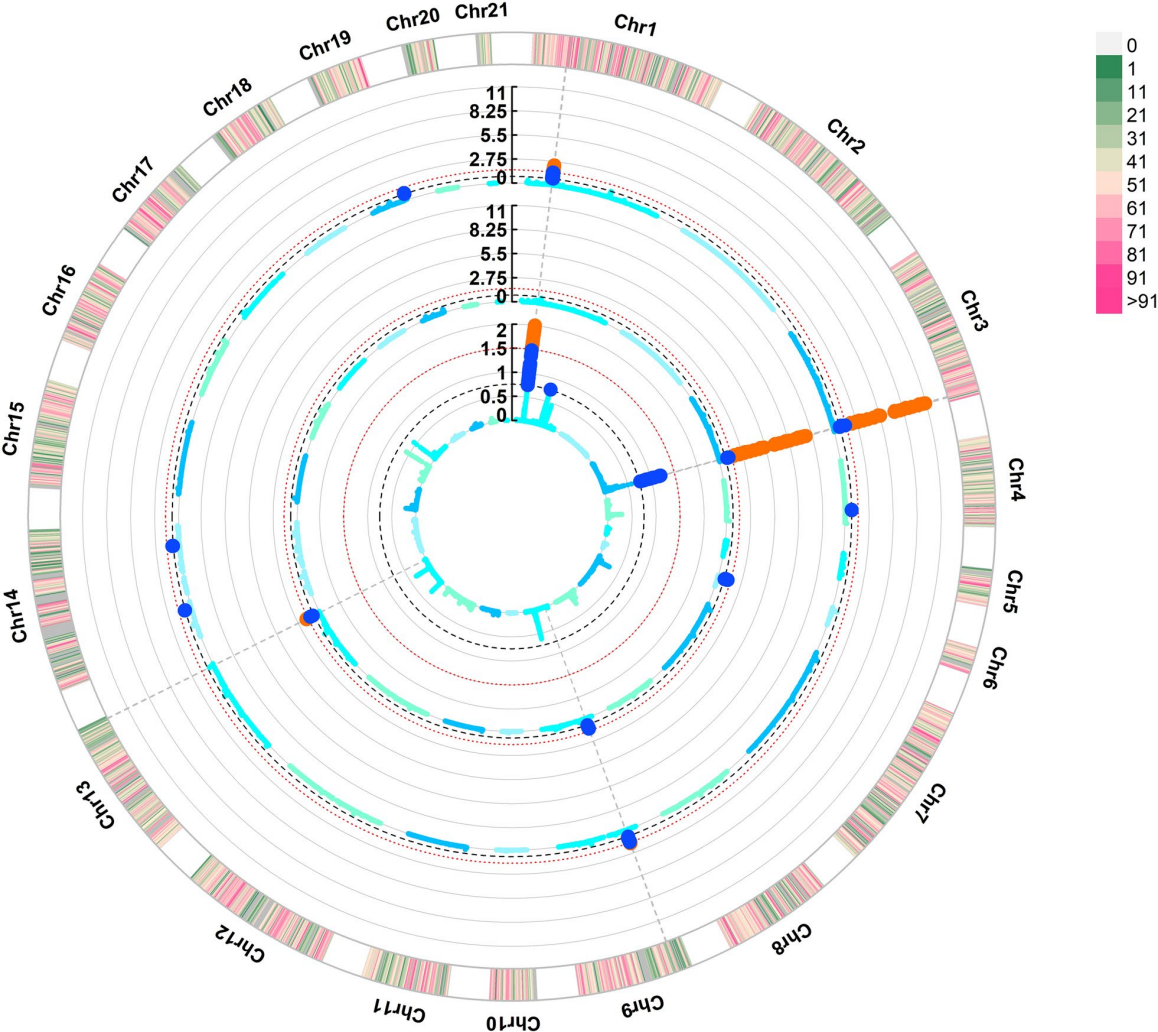
Manhattan plots of genome-wide association studies for intramuscular fat bivariate (inner part), scapular fat univariate (mid part) and scapular fat bivariate (outer part). Each blue dot stands for a genomic window exceeding a 0.75% genetic variance threshold, and each orange dot stands for a window exceeding a 1.50% genetic variance threshold

**Fig. 2:**
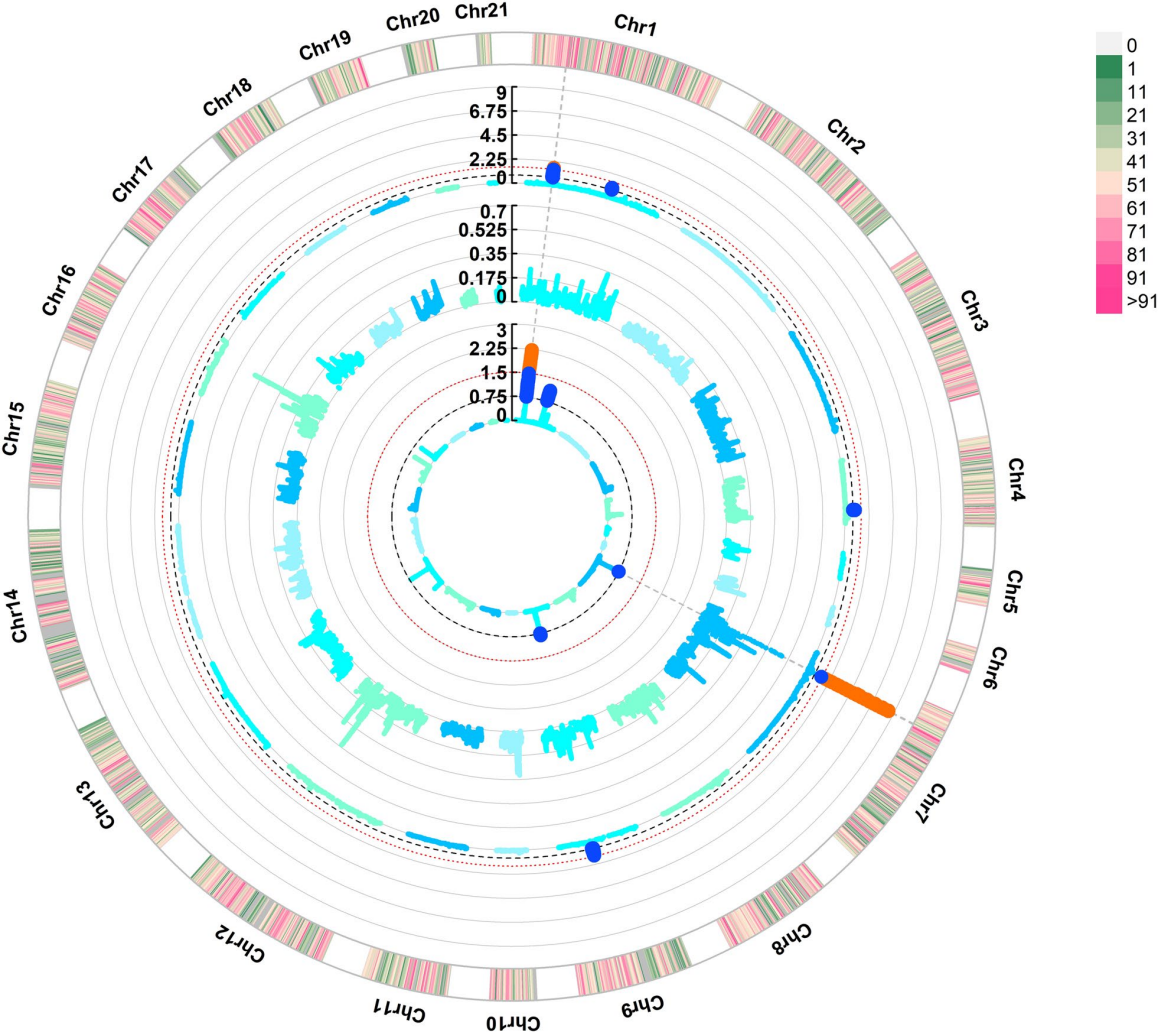
Manhattan plots of genome-wide association studies for intramuscular fat bivariate (inner part), pH univariate (mid part) and pH bivariate (outer part). Each blue dot stands for a genomic window exceeding a 0.75% genetic variance threshold, and each orange dot stands for a window exceeding a 1.50% genetic variance threshold

The region on OCC1 (~ from 35 to 38 Mb) was found to be associated to all traits analysed in this study (except LL*), demonstrating its importance for carcass, fat depot and meat quality traits. This region has also been reported on these lines in a previous selection signature study for IMF [17], and in GWA studies for fatty acid composition [39, 42]. However, this genomic region was not detected neither in the univariate GWAS for SF, weight, and meat quality traits (see for example Fig. 1 for SF and Fig. 2 for pH) nor in the univariate GWAS previously performed for IMF [16]. This represents a good example of how the analysis of related traits under a bivariate model can aid in the detection of associated genomic regions for both traits. The functional annotation of the genes located in this region showed that they were most likely involved in the glucose, growth and lipid metabolisms, whereas the variants found with the WGS pointed to the *SH3GL2*, *CNTLN*, and *BNC2* genes as candidate genes affecting weight, fat depots and meat quality traits. The *SH3GL2* gene is involved in synaptic vesicle endocytosis and lipid binding and modification [43] and, together with *CNTLN*, has been associated with an obesity index in pigs [44]. On the other hand, the expression levels of *BCN2* have been positively correlated with pH in pigs [45]. This region also harbours the *PLIN2* gene, which has a relevant function in lipids metabolism and has been related to IMF content, growth and lean weight in pigs [46, 47]. In these lines, there was one variant detected on this gene, which had the reference allele fixed in the L line, but its frequency in the H line was of 0.6 (i.e., frequency of 0.4 of the alternative allele). Even though this variant did not exceed the threshold assumed (i.e., fixed in one line and a frequency lower than 0.5 in the other), given its relevant function and the evidence in the literature, it still represents a promising candidate to evaluate in future functional analyses.

The pleiotropic region associated to SF and IMF on OCC3 is another interesting example of the advantage of using a pleiotropic GWAS approach. Both univariate and bivariate models were able to detect its association to SF, most likely due to its large effect, explaining over 10% of the genetic variance in both cases (Fig. 1). However, this region was not detected in the univariate analysis for IMF [16] but only in the bivariate GWAS, explaining 1.21% of its genetic variance, which was consistent with the high correlation found between these traits in this window (0.92). This region was also associated with the fatty acid composition in these lines, especially C14:0, C16:0, C18:0, SFA, C18:1n9, MUFA, C18:2n6, C20:4n6, PUFA and PUFA/SFA, explaining between 0.68 and 6.05% of their genetic variance [42]. A total of 6 allele variants were detected in this region, all of which were located in the *ENSOCUG00000000157* novel gene, which is also known as *ST3GAL1* in other species like humans, poultry, pigs, and mice. This gene is involved in lipid metabolism and its expression has also been associated to IMF deposition in broilers [48]. Further analysis of this region could help determine its potential for marker-assisted selection aimed at reducing fat depot accumulation with minimal impact on intramuscular fat (IMF) content. However, weighting-SNP-based genomic selection methods would be a better option than using marker assisted selection in rabbits, especially for economically relevant traits, e.g. growth rate or litter size [49–51].

Under the assumption of pleiotropic effects, the genomic region on OCC7 (26.7–28.9 Mb) was associated with most of the meat quality traits and showed an extreme association value for pH (Fig. 2). This region was not detected in the corresponding univariate GWAS. Regardless of the large number of genes retrieved, no allele variants were detected in the WGS under the assumed parameters. Nonetheless, it still represents an interesting candidate region to evaluate on posterior functional analyses. Finally, another interesting pleiotropic region for meat quality traits was located at 120–121 Mb on OCC1, which was associated with pH and Cb* and it was only detected in the bivariate analyses. This region has consistently appeared in the previous univariate GWAS analysis for IMF performed on these lines (explaining up to 2.03% of the genetic variance) [16], as well as in the selection signature analysis [17] and fatty acid composition analyses [42]. In this region, 4 allele variants were detected, which were located on the novel gene *ENSOCUG00000036784* and on *MAML2*. No evidence was found regarding their involvement in meat quality traits like those discussed in this paper. However, the *MAML2* is a transcription factor that could be affecting several pathways, and has been related to other relevant production traits, like feed efficiency in pigs [52], thus representing an interesting candidate gene for further studies.

## Conclusions

Animal breeding programmes are based on multiple trait selection. In this study, bivariate models assuming pleiotropic effects allowed us to identify relevant genomic regions simultaneously associated with IMF and weight, fat depot and meat quality traits. Additionally, the whole genome sequencing performed allowed us to identify candidate genes with putative functional mutations affecting the mentioned traits. Altogether, our results can provide relevant insights into the correlations and relationships between the traits at the genomic level, which could improve the genomic selection potential. The main pleiotropic region was found on OCC1 between 35.4 Mb and 38.2 Mb, which was associated to almost all analysed traits, with the only exception of the LL* colour parameter. The variants found with the WGS pointed to *PLIN2*, *SH3GL2*, *CNTLN*, and *BNC2* as the main candidate genes in this region affecting weight, fat depots and meat quality traits. Other relevant pleiotropic regions were found on OCC3 between 148.79 and 151.57 Mb, showing the highest association value with SF, and on OCC7 between 26.7 and 28.9 Mb, with the highest association with pH. The allele variants on OCC3 suggested the novel gene *ENSOCUG00000000157* as the main candidate gene affecting fat deposition, whereas on OCC7 there were no allele variants segregating differently between the lines in the identified regions.

## Supplementary Information

Additional file 1: Table S1. Genes retrieved from the genomic regions associated with weight, fat and meat quality traits identified with the bivariate approach. Complete list of genes found in the associated genomic regions to weight, fat and meat quality traits identified in the bivariate genome-wide association analyses

Additional file 2: Table S2. Allele variants found in the genomic regions associated to weight, fat, and meat quality traits. Complete list of allele variants found in the associated genomic regions to weight, fat and meat quality traits identified in the bivariate genome-wide association analyses

Additional file 3: Table S3. Relevant allelic variants located in the genomic regions associated to weight traits. The allelic variants considered relevant were those that were fixed in one line and segregating at an allele frequency lower than 0.5 in the other, and whose alleles were segregating at opposite frequencies between lines

Additional file 4: Table S4. Relevant allelic variants located in the genomic regions associated to fat depot traits. The allelic variants considered relevant were those that were fixed in one line and segregating at an allele frequency lower than 0.5 in the other, andw hose alleles were segregating at opposite frequencies between lines

Additional file 5: Table S5. Relevant allelic variants located in the genomic regions associated to meat quality traits. The allelic variants considered relevant were those that were fixed in one line and segregating at an allele frequency lower than 0.5 in the other, and whose alleles were segregating at opposite frequencies between lines

## Data Availability

The datasets used and analysed during the current study are available from the corresponding author on reasonable request. Genomic data of the ninth generation of intramuscular fat lines were deposited in Figshare Repository (10.6084/m9.figshare.9934058.v1).
